# A lesson learnt: retrospection in a case of pilomatricoma mimicking as parotid neoplasm

**DOI:** 10.1590/S1679-45082015AI3096

**Published:** 2016

**Authors:** Mainak Dutta, Indranil Chatterjee

**Affiliations:** 1 Department of Otorhinolaryngology and Head-Neck Surgery, Medical College and Hospital, Kolkata, India.; 2 Department of Pediatric Surgery, Medical College and Hospital, Kolkata, India.

**Figure 1:**
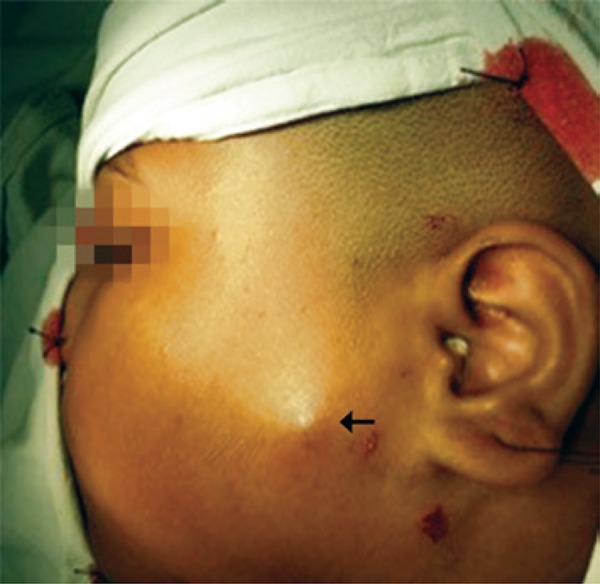
The pre-operative image shows a diffuse swelling in the left pre-auricular region producing an impression of a parotid mass. Note the “tent sign” (arrow). The skin appears taut and stretched. On careful examination, a faint bluish tinge over the lesion could be seen

**Figure 2:**
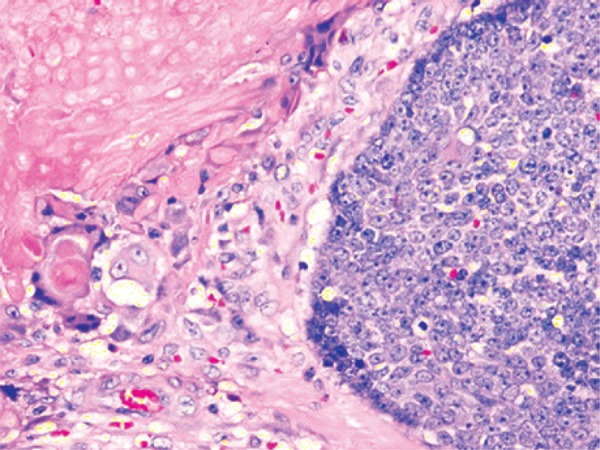
Histopathology shows two predominant cell population of pilomatricoma – the basophilic basaloid cells with scanty cytoplasm, prominent nuclei and indistinct borders, and the eosinophilic anucleated “ghost cells” with relatively distinct borders, and with a transitional zone in between. As the tumor ages, the basaloid cells undergo apoptosis and become “ghost cells”. Hematoxylin-eosin x 400

A 10-year-old girl presented with a painless, slow-growing swelling on left pre-auricular region that was noticed for 2 years (Figure 1). The swelling was bosselated, non-tender, firm-to-hard on palpation, had poorly-defined margin, and measured approximately 3.5cm x 3.0cm. In addition, it had restricted mobility, and seemed to be of parotid origin, with overlying skin apparently stretched and fixed. The ultrasonography was unable to delineate the depth and confirm the involvement of the parotid, although fine needle aspiration cytology (FNAC) suggested pleomorphic adenoma. For this reason, we decided to perform superficial parotidectomy. The histopathology of the lesion diagnosed pilomatricoma, which is an uncommon, benign ectodermal tumor of dermis/subdermis. Pilomatricoma constitutes a pluripotent cell expression in the germinal matrix center of hair follicles with differentiation into cortical cells, and the main relevance of its clinical presence is its potential to be misinterpreted, resulting in unnecessary aggressive interventions. It is often a diagnosis of retrospection. Some large series reported correct pre-operative diagnosis at 1.1 to 29%.^1,2^ A careful re-examination of pre-operative image revealed a subtle bluish tinge, which was not observed presumably because of the dark-skinned complexion, and the characteristic “tent sign”^3^perceptible through the already-stretched and bosselated skin surface (Figure 1). Fine needle aspiration cytology is imperative for the assessment but is often erroneous^4^ due to inadequate, non-representative sampling from a lesion with complex cell contents. In a typical example, the basophilic basaloid cells with scanty cytoplasm and indistinct borders encase the anucleated cytoplasm-rich eosinophilic “ghost/shadow cells” (Figure 2) with areas of keratinization, giant-cells and dystrophic calcification in the matrix. As the tumor gets older, the central “ghost cells” increase at the expense of the peripheral basophilic cells. Thus, FNAC might have more basophilic cells in the lesion in early stages while keratinocytes might predominate in later stages, so that the lesion can be misrepresented as malignant in both stages.^4^ The problem is complicated by the fact that the parotid region is one of the most common topographic areas with involvement of head and neck pilomatricomas,^1,5-7^ which also increase the dilemma of clinicians and pathologists because pilomatricoma is seldom considered as a differential diagnosis for a mass lesion involving this region. In fact, some reports on FNAC from such lesions had suggested parotid neoplasm,^5^ or even metastatic malignant deposits.^6^ Because surgeons relying greatly on cytology in clinically suspected parotid tumors, such approach have resulted in more aggressive surgical treatment.^5,6,8^

The surgical dissection revealed adhesion with overlying skin and superficial lobe of parotid suggesting pathologic tumor adhesion. A pilomatricoma should be surgically excised, but often at the cost of the overlying skin in large tumors since it is a lesion of the dermis/subdermis. A fibrous layer separates the tumor from epidermis and makes the skin taut, giving an impression of adhesion in pre-operative computed tomography scan^8^ and in the surgery. In our case, the tumor was relatively large, and we dissected it from the skin without a surgical plane. Because of the likelihood for recurrence, we decided to keep the patient under close follow-up. Ultrasonography can be a cost-effective modality to delineate tissue plane in lesions of the parotid region especially in children. In addition, it also constitutes a non-invasive tool and does not require sedation. However, in our case it did not show benefits.

Thus, it is important to emphasize that head and neck surgeons and pediatricians should consider pilomatricoma when diagnosing mass lesions involving the parotid region, especially because such lesions constitute the second most common cutaneous tumor in childhood,^9^ and more than half of them involve the head and neck region.^8^ Proper clinical details with representative aspirate in air-dried smears (ensuring maximum yield of “ghost cells”) would guide cytologists to establish the correct diagnosis. To consider the subtle clinical signs and suggest a review of the smear or re-aspiration can avoid surgical “overtreatment” and unwanted morbidity.
