# Supplementary material for: A lesson learnt: retrospection in a case of pilomatricoma mimicking as parotid neoplasm
Source: Einstein (Sao Paulo). 2016 Jan-Mar;14(1):104–5. doi: 10.1590/S1679-45082015AI3096 (PMC4872925; doi:10.1590/S1679-45082015AI3096)
Supplement: Translation

# Translation: Uma lição aprendida: retrospectiva em um caso de pilomatricoma simulando neoplasia de parótida

**Authors:** Mainak Dutta, Indranil Chatterjee

**Affiliations:** 1 Departamento de Otorrinolaringologia e Cirurgia de Cabeça e Pescoço, Medical College and Hospital, Kolkata, Índia.; 2 Departamento de Cirurgia Pediátrica, Medical College and Hospital, Kolkata, Índia.

Paciente do sexo feminino, 10 anos, apresentando edema de crescimento leve e indolor na região pré-auricular esquerda observado por 2 anos (Figura 1). A lesão era bocelada, indolor, firme na palpação, apresentava margens pouco definidas e media aproximadamente 3,5cm x 3,0cm. Além disso, o edema apresentava mobilidade restrita e parecia ter origem na parótida; a pele subjacente estava aparentemente esticada e fixa. A ultrassonografia, porém, não pôde delinear a profundidade e nem confirmar o envolvimento na parótida, apesar de a citologia aspirativa por agulha fina (CAAF) sugerir adenoma pleomórfico. Por essa razão, decidiu-se realizar parotidectomia superficial. A histopatologia, porém, revelou que a lesão tratava-se de um pilomatricoma; um tumor incomum benigno da derme/subderme e ectoderme. O pilomatricoma é expressão celular pluripotente no centro da matriz germinativa de folículos capilares com diferenciação das células corticais, sendo que a principal relevância de sua presença clínica é seu potencial para interpretação inadequada – com frequência de diagnóstico retrospectivo – resultando em intervenção agressiva desnecessária. Alguns estudos relataram diagnóstico pré-operatório em 1,1 a 29%.^1,2^Uma reanálise cuidadosa da imagem pré-operatória revelou tonalidade azulada sútil não observada, presumidamente, pela característica escurecida da pele, e o característico “sinal da tenda”^3^perceptível devido à superfície cutânea bocelada e esticada (Figura 1). A CAAF é necessária para avaliação, porém frequentemente falha,^4^ em razão de amostragem inadequada e não representativa da lesão com conteúdo celular complexo. Em exemplo típico, células basofílicas basaloides com citoplasma escasso e margens indistintas revestem células fantasmas/sombras anucleadas com citoplasma rico em material eosinofílico (Figura 2), com áreas de queratinização, células gigantes e calcificação distrófica na matriz. Com o envelhecimento do tumor, as “células fantasmas” centrais aumentam, às custas das células basófilas periféricas. Portanto, a CAAF pode ter mais células basófilas em lesão em estágio recente enquanto os queratinócitos podem ser predominantes em estágios mais tardios, sendo interpretada, erroneamente, como tumor maligno em ambos os estágios.^4^ O problema é complicado ainda mais em razão da parótida ser uma das áreas topográficas mais comuns com envolvimento do pilomatricoma na região de cabeça e pescoço,^1,5-7^ que também aumenta o dilema dos clínicos e patologistas já que o pilomatricoma é raramente considerado no diagnóstico diferencial para massa envolvendo essa região. Na verdade, há relatos de que a CAAF dessas lesões sugeriu neoplasia da parótida,^5^ ou mesmo lesões malignas metastáticas.^6^ Em razão dos cirurgiões se basearem amplamente na citologia em casos de suspeita clínica de tumores na parótida, tal abordagem tem resultado em tratamentos cirúrgicos mais agressivos.^5,6,8^

**Figura 1:** Imagem pré-operatória mostrando edema difuso na região pré-auricular esquerda produzindo impressão de massa na parótida. Nota-se “sinal de tenda” (seta). A pele apresenta-se tensa e esticada. Em exame detalhado, pode-se observar uma discreta tonalidade azulada sobre a lesão

**Figura 2:** Histopatologia mostrando as duas populações celulares predominantes do pilomatricoma - células basofílicas basaloides com escassez de citoplasma, núcleos proeminentes e margens indistintas, e as “células fantasmas” eosinofílicas e anucleadas, com margens relativamente distintas, com zona transicional no entremeio. Com o envelhecimento do tumor, as células basaloides sofrem apoptose e transformam-se em “células fantasmas”. Hematoxilina-eosina x 400

A dissecção cirúrgica revelou aderência com tecido subjacente e lobo superficial da parótida, sugerindo aderência tumoral. O pilomatricoma deve ser removido cirurgicamente, porém, em geral, pode comprometer a pele subjacente em grandes tumores, já que se trata de lesão da derme/subderme. Um camada fibrosa separa o tumor da epiderme, fazendo com que a pele fique tensa, dando a aparência de adesão na tomografia computadorizada^8^ no pré-operatório e na cirurgia. Em nosso paciente, o tumor era relativamente grande, sendo dissecado sem delimitação de plano cirúrgico. Em razão da possibilidade de recidiva, decidimos manter o paciente em seguimento. A ultrassonografia pode ser uma modalidade custo-efetiva para delinear o plano do tecido em lesões na região da parótida, especialmente em crianças. Além disso, trata-se de ferramenta não invasiva e dispensa o uso de sedativo. Todavia, em nosso caso, não apresentou benefícios.

É importante enfatizar que cirurgiões de cabeça e pescoço e pediatras devem considerar o pilomatricoma para diagnosticar lesões envolvendo a região parótida, especialmente por essas lesões serem o segundo tumor cutâneo mais comum na infância,^9^além de mais da metade deles afetar a região da cabeça e pescoço.^8^ Detalhes clínicos adequados com esfregaços citológicos representativos (assegurando o achado das “células fantasmas”) devem guiar os citologistas para estabelecer o diagnóstico correto. Considerar os sinais clínicos e sugerir revisão dos esfregaços, ou refazer as aspirações da lesão, podem evitar tratamento cirúrgico excessivo e reduzir possível morbidade.
